# Analysis of Maxillary First Molar Derotation with Invisalign Clear Aligners in Permanent Dentition

**DOI:** 10.3390/life12101495

**Published:** 2022-09-26

**Authors:** Roberta Lione, Valeria Paoloni, Francesca Chiara De Razza, Chiara Pavoni, Paola Cozza

**Affiliations:** 1 Department of Systems Medicine, University of Rome Tor Vergata, 00133 Rome, Italy; 2 Department of Health Sciences, Uni-Camillus Saint Camillus International University, 00131 Rome, Italy

**Keywords:** Class II, aligners therapy, 3D cast analysis

## Abstract

The objective of this study was to examine the distal rotation of mesial rotated maxillary first permanent molars in a sample of Class II dental malocclusion adult patients treated with Invisalign Clear Aligners (CA). Forty patients (20 males, 20 females, 22.4 ± 3.9 years) were included in the study sample (Department of Orthodontics of University of Rome “Tor Vergata”). Inclusion criteria were: Caucasian ancestry, complete permanent dentition with fully erupted upper second molars, Class II molar relationship, absence of tooth or craniofacial anomalies or caries and periodontal diseases. Pre-treatment (T1), post-treatment (T2) digital casts, and final ClinCheck simulation models (T2CC) were analysed. To measure the rotation of maxillary first molars, Henry’s angle (H°) was evaluated. Maxillary first molars with an H° > 11° were considered mesio-rotated (in total 59 teeth). The treatment CA protocol included disto-rotation without distalization movements. At T1, T2 and T2CC five measurements on the collected dental casts were analysed: Henry’s angle (H°); mesial buccal expansion (ME); distal buccal expansion (DE); mesial buccal sagittal (MS); and distal buccal sagittal (DS). A comparison between the results of T2-T1 and T2CC-T2 was performed using a paired *t*-test. The differences between T2-T1 highlighted a significant distal rotation of the maxillary first molars (−7.4°) and an expansion movement of 2.20 mm for ME and 1.50 mm for DE. In the post-treatment, the mesial buccal cusps shifted of 1.0 mm, while the distal buccal cusps showed a distal movement of 0.9 mm. Analysing the H° comparison between T2CC-T2, the difference was −1.1°. The T2CC-T2 comparison in the sagittal plane showed a difference of 0.9 mm for the MS and 0.7 mm for the DS. The accuracy was 82% for molar derotation movement. In conclusion, CA provides the upper arch expansion associated with the upper first molars’ distal rotation. These movements provide 2 mm of improvement in arch perimeter and molar intercuspation.

## 1. Introduction

Correction of a Class II dental relationship cannot be successfully managed without the evaluation of the Class II malocclusion aetiology [1].

It has been reported that mesial palatal rotation of maxillary first permanent molars occurs in 95% of dental Class II-division 1 malocclusion patients and it is assessed in 83% of Class II patients [2,3].

In 2011, Junqueira et al. [4] compared the dental casts of patients in normo-occlusion with subjects in Class II division 1 malocclusion. They found that the Class II division 1 malocclusion group presented with a higher number of mesio-palatal rotations of the upper first molar if compared to the normo-occlusion group.

Furthermore, there is a strong correlation between mesial rotated upper first molar and transversal dento-alveolar discrepancy, indeed in 100% of all maxillary constrictions first molars show mesial rotation [5].

Upper first molar displacement occurs within the shift from mixed to permanent dentition, due to molar mesial movement into the leeway space. What causes the mesial rotation is the maxillary first molar mesiolingual cusp, which occludes in the central fossa of the mandibular first molar as a rotation centre, with the creation of a mesial buccal cusp mesial movement [3]. This induces a decrease of the arch perimeter length and results in a mesial rotation of the whole anterior teeth, with the creation of a Class II dental relationship and an increase of overjet. Any further mesial drift from anterior crowding and/or arch constriction further exacerbates this problem [2].

The correction of molar rotation not only helps to classify the molars into a Class I relationship, but concomitantly allows subsequent distalization and Class I correction [5].

In the literature, it is reported that it is mandatory to correct the rotation of Upper First Molars to obtain adequate space in the upper arch, which is necessary to achieve a correct occlusion [6,7,8].

However, in the literature only a few studies have focused on the strategies to correct the upper first molar mesial rotation using Clear Aligners (CA), in particular in permanent dentition. 

Lombardo et al. (2017) [9] analysed the predictability of orthodontic movements using F22 Aligners. They examined a sample of 16 patients in permanent dentition and found out that first molar rotation correction occurred with a percentage of accuracy around 79%.

In 2020, Morales-Burruezo et al. [10] examined a sample of 114 patients to investigate the accuracy of arch expansion with Invisalign Clear Aligners, and found a predictability of 79.1% for first molars rotational movement.

A recent study of Lione et al. [11] analysed the derotational movement of upper first mesial rotated molars in 36 Class II edge-to-edge malocclusion growing patients. They found out that CA are effective at allowing upper distal molar rotation with a predictability of 60% in growing patients.

The objective of this study was to evaluate the movements of mesial rotated upper first permanent molars in a sample of Class II dental malocclusion adult patients in treatment with Invisalign Clear Aligners (CA).

## 2. Materials and Methods

This investigation was authorized by the University of Rome “Tor Vergata” ethical committee (Protocol number: 163/20). Patients were conscious of the project design and their informed consent was collected before the beginning of the therapy.

A sample of 40 patients (20 males, 20 females, mean age 22.4 ± 3.9 years) treated with Invisalign System Clear Aligners (CA) were consecutively enrolled at the Department of Orthodontics of the University of Rome “Tor Vergata” from October 2020 to November 2021.

### 2.1. Inclusion and Exclusion Criteria

Patients were chosen according to these inclusion criteria: Caucasian ancestry, permanent dentition with fully erupted second molars, Class II molar occlusal relationship, compliance with CA. Exclusion criteria applied were multiple and/or advanced caries, supernumerary teeth or tooth agenesis, necessity of extraction treatment, previous cleft lip and/or palate anamnesis, or periodontal diseases.

### 2.2. Therapeutic and Measurement Protocol

Pre-treatment (T1) and post-treatment (T2) digital casts were taken for all subjects through the intraoral scanner iTero^®^ Orthodontic ver. 5.2.1.290 (Align Technology Inc., Santa Clara, CA, USA). The final ClinCheck representation was also collected to state the correspondence of the 3D model (T2CC) compared to the movements occurring in the T2 casts.

**Figure 1:**
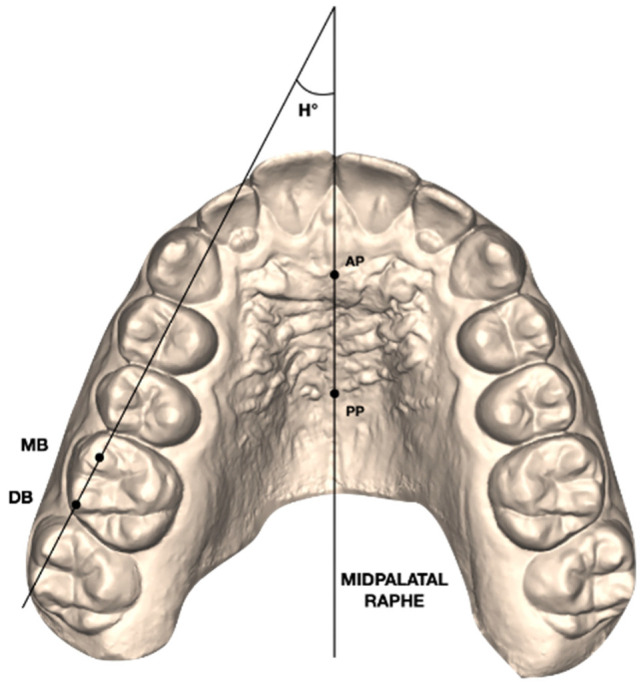
Henry’s angle (H°): angle formed between the midpalatal raphe (AP-PP) and a line passing by the mesial buccal (MB) and distal buccal (DB) cusps of the upper first molar.

On the T1 digital models, the maxillary permanent right and left first molars’ rotations were evaluated by the Henry’s angular measurement [2] (Figure 1).

All the maxillary permanent first molars with a Henry’s angle of more than 11 degrees were considered mesio-rotated and collected in the investigation, acquiring a sample of 59 teeth.

For each patient, the ClinCheck was programmed using ClinCheck software with a programmed distal rotation protocol following the Rickett’s line [12]. 

A mesial-out rotation of maxillary first molars (2 degrees for each aligner) was programmed. Simultaneously to the mesial-out rotation, the upper arch expansion was also planned, while no distalization movements were programmed.

Optimized expansion support attachments and optimized retention attachments were positioned by the software on the lateral-posterior segment of the upper arch.

Patients were told to wear the aligners for the whole day, except for meals and for tooth brushing. The patient changed their aligners every 7 days [13], and every four aligners’ clinicians scheduled a follow-up to check the aligner fitting and the correct attachments placement.

The mean number of aligners was 32 for the maxillary arch and the average time between the initial and final digital scan was 11.9 ± 2.4 months.

Patients became aware that they took part in a research study at the delivery appointment, and reliable compliance reporting was challenging. A single investigator carried out follow-up interviews with each patient to state the level of his/her compliance and noted that in their clinical diary.

The patients’ collaboration was measured on a three-point (poor, moderate, good) Likert-type scale [14]: low compliance was stated when the patient wore the aligners for less than 16 h per day; moderate from 16–20 h per day; good when the patient wore the aligner full time.

**Figure 2:**
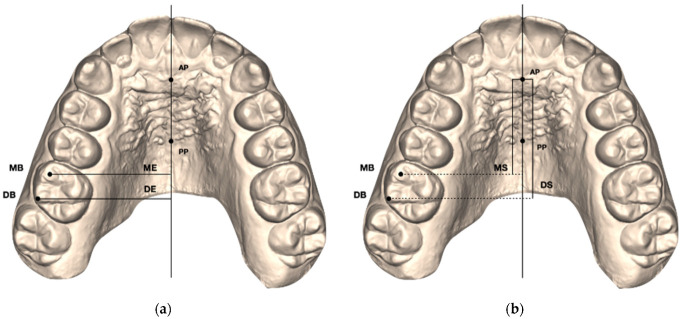
(**a**) Linear measurements of the expansion movements (ME, DE) of the maxillary first molar mesial buccal (MB) and distal buccal (DB) cusps. (**b**) Linear measurements of the sagittal movements (MS, DS) of the maxillary first molar mesial buccal (MB) and distal buccal (DB) cusps.

Maxillary digital dental casts were exported in .stl format. For each of the 59 upper permanent first molars included in the study, the following measurements were performed at T1, T2, and T2CC using Viewbox 4 (dHAL software, Kifissia, Greece):

- Henry’s angle (H°): angle between the midpalatal raphe (identified by an anterior AP and a posterior PP point along the mid-palatal raphe) and the line passing through the mesial buccal (MB) and distal buccal (DB) cusps of the maxillary first molar [2]; (Figure 1);
- Mesial buccal Expansion (ME): linear distance between the mesial buccal cusp of the maxillary first permanent molar and midpalatal raphe (AP-PP) measured on each side [11] (Figure 2a);
- Distal buccal Expansion (DE): linear distance between the distal buccal cusp of the maxillary first permanent molar and midpalatal raphe (AP-PP) measured on each side [11] (Figure 2a);
- Mesial buccal Sagittal (MS): linear distance between the middle point of the first palatal rugae (AP) and the projection of the mesial buccal cusp of the maxillary first permanent molar on the midpalatal raphe measured on each side [11] (Figure 2b);
- Distal buccal Sagittal (DS): linear distance between the middle point of the first palatal rugae (AP) and the projection of the distal buccal cusp of the maxillary first permanent molar on the midpalatal raphe measured on each side [11] (Figure 2b).

## 3. Statistical Analysis

In a pilot study, 12 upper first permanent molars were used to calculate the sample size, which indicated the need for approximately 45 teeth to estimate Henry’s angle with a 95% confidence interval (CI), a minimum difference of 2.3° and a standard deviation (SD) of 3.5°, with a power of 80%.

A unique operator (F.C.D.R.) performed all the measurements. In order to test the intra-examiner reliability, the same operator measured the entire sample again 2 weeks after the first assessment. The reliability of the measure was assessed by means of an interclass correlation coefficient (ICC).

Sample normality was tested by the Shapiro–Wilk test.

Since the data were normally distributed, the paired *t*-test was chosen to analyse the T2-T1 and the T2CC-T2 differences. The level of significance was set at 5%.

The Statistical Package for the Social Sciences (SPSS) software, version 18.0 (IBM Corp, Chicago, IL, USA) was used to compare data. 

## 4. Results

Among the 80 collected molars from the 40 patients, 59 presented increased mesial rotation according to Henry’s angle of more than 11 degrees. There was no statistically significant difference in the distribution of the mesial-rotated molars between male and female. 

The evaluation of subjects’ compliance (use of CA) highlighted that cooperation was good/moderate in all of them.

The ICC test showed a score of 0.97 for the angular measurement and 0.96 for the linear measurements.

### 4.1. Pre-Treatment (T1) and Post-Treatment (T2) Comparisons

The differences between T1 data and T2 outcomes are shown in Table 1. Statistically significant differences for all the performed measurements were found.

The comparison between T2-T1 H° highlighted a maxillary first molars’ disto-rotation of −7.4° (mean H° T1: 18.4° ± 4.9°; mean H° T2: 11° ± 3.2°; *p* < 0.001). The mean transversal expansion was 2.2 mm for the mesial buccal cusps (mean ME T1: 25.6 ± 3.5 mm; mean ME T2: 30.6 ± 6.2 mm; *p* < 0.001) and 1.5 mm for the distal buccal cusps (mean DE T1: 27.6 ± 4.6 mm; mean DE T2: 33.5 ± 6.1; *p* < 0.001). On the sagittal plane, the mesial buccal cusps showed a sagittal shift of 1.0 mm at T2 (mean MS T1: 29.3 ± 2.2 mm; mean MS T2: 30.3 ± 2.5 mm; *p* < 0.001), while the distal buccal cusps moved 0.9 mm distally (mean DS T1: 24.3 ± 2.1 mm; mean DS T2: 25.2 ± 2.5 mm; *p* < 0.001).

### 4.2. Post-Treatment (T2) and Final ClinCheck (T2CC) Comparisons

Table 2 describes the differences between T2CC-T2. The accuracy of the Invisalign ClinCheck software was determined at the end of the treatment by a comparison between the final movements achieved in the digital models (T2) and the planned one (T2CC). 

Regarding molar rotations, a statistically significant difference was found, meaning that the planned change was not fully comparable with the results achieved. The mean difference between T2CC-T2 was 2.01° (mean H° T2: 11.06° ± 3.16°; mean H° T2CC: 9.05° ± 2.4°; *p* < 0.01). The molar derotation movement shows a percentage predictability of the programmed movements about 82%.

Comparable results were obtained for the measurements on the sagittal plane, showing a mean difference of 0.9 mm for the mesiobuccal cusps (mean MS T2: 30.3 ± 2.5 mm; mean MS T2CC: 31.2 ± 2.3 mm; *p* > 0.5) and 0.7 mm for the distobuccal cusps (mean DS T2: 25.2 ± 2.5 mm; mean DS T2CC: 25.9 ± 2.2 mm; *p* > 0.5). The expansion movements showed high predictability for both the mesiobuccal and distobuccal cusps. The mean difference between T2CC-T2 was 0.1 mm for both ME and DE, meaning no statistically significant changes. 

Table 3 shows the movement percentage accuracy, measured by comparing the final upper first molar position achieved with the planned one. Upper first molar derotation showed a predictability of 82%, expansion had a percentage of 99% and sagittal movement of 97%.

## 5. Discussion

This prospective study evaluated the modifications induced by treatment with CA on maxillary molar position and aimed to assess the accuracy of digital planning in a group of adult patients with Class II malocclusion and mesio-rotated upper molars.

Plenty of investigations [2,3,4,5,6,7,8,11,15,16,17,18,19,20,21,22,23,24,25,26,27,28,29,30] stated a high percentage of mesial-rotation of upper first molars in Class II malocclusion. In 2019, de Oliveira-Viganò et al. stated that dental Class II show the highest levels of mesio-palatal rotation of the upper first molars [15].

Because of its trapezoidal-shaped form and its triple roots, the upper first molar shows a mesiolingual rotation on its long axis. Maxillary first molar mesiopalatal cusp is a pivotal axis, performing as a centre of rotation [6]. 

The primary goal of digital treatment planning is to recreate a functional intercuspation by expanding and derotating at the same time [16]. For this reason, it is important to carefully diagnose this concern and, during the digital planning, to plan the upper first molar’s mesial-out buccal rotation, to make possible its distal rotation on the mesiopalatal cusp. 

Our results showed an accuracy of 99% for the expansion movement. Several studies [17,20,21,22,23,24,25,26,27,28,29] focused on the expansion movement with Clear Aligners, analysing the molar transversal movement predictability.

In 2017, Zhao et al. [23] examined a sample of 31 adult patients and evaluated the transversal changes between pre-treatment and post-treatment by measuring upper intermolar width and molar buccal inclination. They found out that the predictability of molar expansion was 29% and affirmed that posterior teeth occurred principally for vestibular crown tipping. 

Houle et al. [24] considered a sample of 64 patients in permanent dentition. They measured maxillary posterior width and found that upper transversal change predictability was about 72.8%.

In 2020, Zhou et al. [25] analysed a predictability of expansion in a sample of 20 adult patients and assessed a percentage of bodily expansion movement about 36.35% on upper first molars, stating that aligners could increase the arch width achieved by tipping movement.

In 2021, Riede et al. [26] analysed molar expansion movement in a sample of 30 patients and found 45% predictability in transversal change. Focusing on their sample, patients showed a large deviation of age (from 15 to 64 years old) and presented important transversal discrepancies between maxilla and mandibula. 

All of these cited studies did not consider the rotational movement of Upper First Molars as an improvement tool to make the expansion with Clear Aligners more predictable. Furthermore, they focused on measuring the transversal width at gingival level. This measurement should be not considered as a valid method, because during ClinCheck’s planning process the gum is automatically removed from the digital model by the software—as it determines the treatment parameters and protocols—and is randomly positioned without applying any specific criteria. For this reason, gingival data cannot be correct and may vary from one ClinCheck simulation to another, which means that the virtual gingiva will be not positioned in a standard way.

Our results strongly agreed with those obtained by Vidal Berdardez et al. in 2021 [27] whose assessment found a coronal expansion width of about 98.32%. 

Furthermore, our results proved that there were statistically significant differences between T2-T1 maxillary first molar derotation of about 6 degrees. 

We analysed mildly mesial rotated upper molars in a Class II adult sample. Our treatment protocol required molar disto-rotation without any distalization. 

Moreover, derotation of the maxillary first molar with the application of a simultaneous expansion movement can provide multiple forces’ resistance that migrates from the molar centre of resistance toward the distal marginal ridge, thus allowing a distal movement of the rotation centre [10]. According to our results, an orthodontic molar derotation of about 6 degrees led to a mean gain of arch space of about 2 mm. 

Braun et al. [28] stated an amount of gain in the arch perimeter of 2.5 mm associated with an upper molar derotation of about 20 degrees. The gain in arch perimeter occurred anteriorly to the derotated tooth: the buccal cusps shifted distally by 2 mm with an improvement in the molar–occlusal relationship. [28]

Our results strongly agreed with other studies that analysed the rotational movement during expansion therapy with Clear Aligners.

Lombardo et al. (2017) [9] analysed the predictability of orthodontic movements using F22 Aligners. They examined a sample of 16 patients in permanent dentition and found that first molar rotation occurred with an accuracy of around 79%.

In a recent study, Haouili et al. [31] stated an upper first molars mesio-rotation of 42.9%. The difference in percentages could be explained with a different maxillary arch expansion staging during the ClinCheck planning, because is essential to provide arch expansion in order to recover further space and increase the accuracy of distal rotation movement. 

In 2020, Burruezo et al. [10] examined the transversal changes of 114 adult patients. They analysed not only upper first inter-molar width (predictability of 79.1%) but also first molar rotation movement, programmed contemporarily to upper first molar expansion. In their study, upper molar rotation had an accuracy percentage of 80%. These results agreed with our findings, showing that it is crucial to program a correct molar derotation when planning expansion movements.

In a recent study, D’Antò et al. (2022) [29] analysed movement accuracy with Clear Aligners and stated that upper first molars’ rotation showed a high predictability of success. 

In 2021, Lione et al. [11] stated a good accuracy of the molar programmed expansion and distal rotation with CA in mixed dentition, with a predictability of 60%. However, in this study they only considered a sample of children with mixed dentition, without fully erupted upper second molars.

Our data also highlighted that Invisalign CA allows the achievement of distal rotation combined with transverse expansion with a predictability of 82%.

The different percentages of our investigation and the previous one could be explained by the mixed dentition of growing patients presenting a shorter height of molar clinical crowns and a lower gingival margin position with respect to the dental crown [32]. Furthermore, staging and attachments used in growing patients are different when compared with adult patients.

Our investigation found that there were differences between T2 achieved rotation and the T2CC programmed one. ClinCheck software overestimated the results obtained in molar derotations planning. To test the accuracy of ClinCheck planning, measurements taken at T2 were analysed in comparison with outcomes planned for the end of the first phase of aligners (T2CC) with a predictability of 82%. These results agreed with those obtained by other studies [9,10,11,12,13,14,15,16,17,18,19,20,21,22,23,24,25,26], reporting that the ClinCheck prospected a greater distal rotation than realized and which is necessary to provide a further correction of mesial rotation movement during the refinement additional phase.

The main limitations of the present study are represented by the small sample size and its short-term aspect. We recommend other investigations with a greater sample size to test the reproducibility, and it would be also mandatory to analyse the stability of the outcomes in the long term.

## 6. Conclusions

Subjects in permanent dentition with dental Class II frequently show a mesial rotation of maxillary first molars. Of 80 molars, 59 showed an increased mesial rotation. Distal rotation of maxillary first molars is a movement that involves the expansion and distal shift of buccal cusps. Although this movement is difficult, CA are effective at allowing maxillary distal molar rotation with an observed 82% predictability. The orthodontic correction of mesial rotation guaranteed 2 mm of gain in the arch perimeter and an improvement in molar relationships, with the movement of transversal expansion showing high accuracy.

## Figures and Tables

**Table 1:** Statistical analysis of T2-T1 changes (paired *t*-test).

Measurements	Pre-Treatment T1 (*n* = 59)		Post-Treatment T2 (*n* = 59)			
	Mean	SD	Mean	SD	Diff	*p* Value
Henry’s angle (H°)	18.43	4.91	11.06	3.16	−7.37	***
Mesial buccal Expansion (ME)	25.6	3.50	30.62	6.20	2.2	***
Distal buccal Expansion (DE)	27.6	4.6	33.51	6.10	1.5	***
Mesial buccal Sagittal (MS)	29.3	2.2	30.3	2.5	1.0	***
Distal buccal Sagittal (DS)	24.3	2.1	25.2	2.5	0.9	***

SD: standard deviation; diff: mean difference; *** = *p* < 0.001.

**Table 2:** Statistical analysis of T2CC-T2 results (paired *t*-test).

Measurements	Post-Treatment T2 (*n* = 59)		T2 ClinCheck (*n* = 59)			
	Mean	SD	Mean	SD	Diff	*p* Value
Henry’s angle (H°)	11.06	3.16	9.05	2.41	2.01	**
Mesial buccal Expansion (ME)	30.62	6.20	30.71	0.7	0.1	NS
Distal buccal Expansion (DE)	33.51	6.10	33.4	0.8	0.11	NS
Mesial buccal Sagittal (MS)	30.3	2.5	31.2	2.3	0.9	NS
Distal buccal Sagittal (DS)	25.2	2.5	25.9	2.2	0.7	NS

SD: standard deviation; diff: mean difference; ** = *p* < 0.01.

**Table 3:** Predictability of the movements (T2-T2CC).

Variable	Percentage %
Derotation	82%
Expansion	99%
Sagittal Movement	97%

## Data Availability

The data presented in this study are available on request from the corresponding author.

## Abbreviations

CA: Clear Aligners; H°: Henry’s angle; MB: Maxillary First Molar Mesial buccal cusp; DB: Maxillary First Molar Distal buccal cusp; ME: Mesial buccal expansion; DE: Distal buccal expansion; MS: Mesial buccal sagittal; DS: Distal buccal sagittal; T1: pre-treatment phase; T2: post-treatment phase; T2CC: final ClinCheck representation; IC (confidence interval); SD (standard deviation); diff: mean difference; ICC (interclass correlation coefficient).
